# Active Zone Material-Directed Orientation, Docking, and Fusion of Dense Core Vesicles Alongside Synaptic Vesicles at Neuromuscular Junctions

**DOI:** 10.3389/fnana.2018.00072

**Published:** 2018-09-13

**Authors:** Jae H. Jung, Joseph A. Szule, Kylee Stouder, Robert M. Marshall, Uel J. McMahan

**Affiliations:** ^1^Department of Biology, Texas A&M University, College Station, TX, United States; ^2^Department of Neurobiology, Stanford University School of Medicine, Stanford University, Stanford, CA, United States

**Keywords:** synapse, neuromuscular junction, dense core vesicle, synaptic vesicle, vesicle docking, active zone material, electron tomography

## Abstract

Active zone material is an organelle that is common to active zones along the presynaptic membrane of chemical synapses. Electron tomography on active zones at frog neuromuscular junctions has provided evidence that active zone material directs the docking of synaptic vesicles (SVs) on the presynaptic membrane at this synapse. Certain active zone material macromolecules connect to stereotypically arranged macromolecules in the membrane of undocked SVs, stably orienting a predetermined fusion domain of the vesicle membrane toward the presynaptic membrane while bringing and holding the two membranes together. Docking of the vesicles is required for the impulse-triggered vesicle membrane-presynaptic membrane fusion that releases the vesicles’ neurotransmitter into the synaptic cleft. As at other synapses, axon terminals at frog neuromuscular junctions contain, in addition to SVs, vesicles that are larger, are much less frequent and, when viewed by electron microscopy, have a distinctive electron dense core. Dense core vesicles at neuromuscular junctions are likely to contain peptides that are released into the synaptic cleft to regulate formation, maintenance and behavior of cellular apparatus essential for synaptic impulse transmission. We show by electron tomography on axon terminals of frog neuromuscular junctions fixed at rest and during repetitive impulse transmission that dense core vesicles selectively dock on and fuse with the presynaptic membrane alongside SVs at active zones, and that active zone material connects to the dense core vesicles undergoing these processes in the same way it connects to SVs. We conclude that undocked dense core vesicles have a predetermined fusion domain, as do undocked SVs, and that active zone material directs oriented docking and fusion of these different vesicle types at active zones of the presynaptic membrane by similar macromolecular interactions.

## Introduction

Active zones along the presynaptic plasma membrane of chemical synapses are characterized by one or more dense aggregates of proteinaceous macromolecules (variously shaped filaments) called active zone material (AZM), which are attached to the presynaptic membrane and extend into the cytosol. The size, shape, and distribution of AZM can vary from one synaptic type to another within an animal species and for the same synaptic type between species (Palade, 1954; Palay, 1956; Zhai and Bellen, 2004). Despite such variation, synaptic vesicles (SVs), which contain small molecule neurotransmitters, move toward and dock on, i.e., are held in contact with, the presynaptic membrane alongside AZM. There, the membrane of the SVs fuses with the presynaptic membrane upon arrival of a nerve impulse, releasing the neurotransmitters into the synaptic cleft to act on the postsynaptic cell. At various synapses where active zones have been examined by tilt-axis transmission electron tomography on tissue sections, which is currently the only method available for imaging individual AZM macromolecules *in situ*, the macromolecules link the membranes of docked SVs to the presynaptic membrane (Harlow et al., 2001; Zampighi et al., 2006, 2008, 2011; Siksou et al., 2007, 2009; Nagwaney et al., 2009; Fernandez-Busnadiego et al., 2010, 2013; Jiao et al., 2010; Stigloher et al., 2011; Burette et al., 2012; Szule et al., 2012; Imig et al., 2014; Cole et al., 2016; Jung et al., 2016). Several lines of evidence make it seem likely that the AZM macromolecules include proteins shown by biochemistry to regulate both the docking and fusion of SVs (Harlow et al., 2001; Szule et al., 2012; Jung et al., 2016).

While conventional two-dimensional (2D) transmission electron microscopy offers little measurable spatial information in the depth axis of a tissue section, tilt-axis electron tomography provides quantifiable spatial data in all three axes by using a series of 2D images of a section taken at different degrees of tilt to generate a three-dimensional (3D) reconstruction (Frank, 1992). Structures of interest in a reconstruction can then be examined at 2 nm - 3 nm spatial resolution in serial 2D virtual slices through the volume, or as 3D surface models after utilizing the virtual slices to segment them from the volume (Ress et al., 1999, 2004).

Using electron tomography on individual tissue sections from frog neuromuscular junctions, we found that the simply arranged AZM on the axon terminals’ presynaptic membrane is composed of an organized network of structurally distinct classes of thin, elongate macromolecules (Harlow et al., 1998, 2001; Szule et al., 2012). Multiple members of certain classes connect to the membrane of each docked SV in an ordered arrangement that is stereotypic from one docked SV to the next. The lumen of SVs also contains an interconnected assembly of elongate macromolecules (Harlow et al., 2013). The assembly radiates from near the center of the lumen, and it has a chiral shape that is stereotypic for both docked and undocked SVs. Certain of its components, called nubs, reach the SV membrane where they connect to and anchor macromolecules that span the membrane. From the orientation of the luminal assembly’s shape in docked SVs and in undocked SVs prior to and during the process of docking, it appears that for docking to proceed, undocked SVs must rotate in a way that aligns a specific set of transmembrane macromolecules and their nubs with AZM macromolecules. Stable connections then form between the transmembrane macromolecules and the AZM macromolecules in successive steps beginning with those AZM macromolecules farthest from the presynaptic membrane, which ultimately brings the SV membrane into contact with the presynaptic membrane and holds it there. Accordingly, it is reasonable to conclude that the fusion domain on docked SVs is predetermined on undocked SVs, and it is the role of AZM macromolecules by their pairing with and connecting to the stereotypically arranged transmembrane macromolecules and their nubs in undocked SVs not only to direct the undocked SVs to docking sites on the presynaptic membrane, but also to stably orient the fusion domain of the SV membrane toward the presynaptic membrane during this process. The orientation of a predetermined fusion domain on a SV membrane for contact with the presynaptic membrane may provide advantages for normal SV membrane-presynaptic membrane fusion, the effectiveness of the SV content released into the synaptic cleft and/or the efficient retrieval of SV membrane from presynaptic membrane after fusion has occurred (Harlow et al., 2013).

In addition to their connections to AZM macromolecules, docked SVs are connected to several elongate non-AZM macromolecules. They link the docked SVs to nearby undocked SVs and other organelles, and they are grossly similar in appearance to macromolecules that connect undocked SVs to each other and other organelles throughout the axon terminals of synapses across species (Llinas et al., 1985; Landis et al., 1988; Hirokawa et al., 1989; Feng et al., 2002; Szule et al., 2012; Cole et al., 2016). Nubs of the luminal assembly are paired with and connected by transmembrane macromolecules to the non-AZM macromolecules as they are to AZM macromolecules. While the breaking and making of non-AZM macromolecules must regulate the movement of SVs in general (Llinas et al., 1985), there is no evidence to date indicating that they specifically direct SVs to their docking sites at active zones on the presynaptic membrane as do AZM macromolecules (Szule et al., 2012).

At synapses throughout the nervous system, axon terminals contain, in addition to SVs, vesicles that exhibit a distinctive electron-dense core when stained with heavy metals and viewed by electron microscopy. Typically distributed among SVs, dense core vesicles (DCVs) are greater in diameter and much fewer in number than the SVs. They contain peptides, which are considerably larger than the neurotransmitters in SVs and have different effects on the postsynaptic cell from those of the neurotransmitters (Nitsch and Rinne, 1981; Zupanc, 1996; Ahmari et al., 2000; Hartmann et al., 2001; Shakiryanova et al., 2006; Dieni et al., 2012; Wong et al., 2012). Electrophysiology has provided evidence that impulse activity elicits fusion of the membrane of DCVs with the axonal plasma membrane for the release of their content as it does for the membrane of SVs (Verhage et al., 1991; Hartmann et al., 2001; Matsuda et al., 2009; van de Bospoort et al., 2012). It has been difficult to determine by conventional electron microscopy on tissue sections whether or not DCVs dock on and their membrane fuses with the presynaptic membrane at active zones because of their relative paucity and the lack of reliable depth axis information essential for detailed characterization of their relationships (but see Dickinson-Nelson and Reese, 1983). However, cryo-electron tomography on cultured hippocampal synapses has shown that DCVs can fuse with the presynaptic membrane at active zones of such synapses (Tao et al., 2018).

We noticed in our electron tomography reconstructions used for studies on the role of AZM in the docking of SVs at frog neuromuscular junctions (Szule et al., 2012; Jung et al., 2016), that DCVs, which constitute ∼1% of the axon terminal’s total vesicle population at this synapse (Pecot-Dechavassine and Brouard, 1997), selectively dock on and fuse with the presynaptic membrane at the active zones together with the SVs. Here, we document these observations, and we show, in addition, that the same classes of AZM macromolecules that connect in a particular pattern to the membrane of docked and docking SVs also connect in the same pattern to the membrane of docked and docking DCVs. Non-AZM macromolecules also connect to the DCVs in the same way they do to SVs. We show, further, that the core of the DCVs is composed of an interconnected assembly of macromolecules which, while more extensive and more compact than the luminal assembly of SVs, is also connected by nubs to the DCV membrane at sites that are paired with the connection sites of AZM and non-AZM macromolecules. Altogether the results indicate that the AZM directs the orientation, docking and fusion of a predetermined fusion domain on DCVs at the presynaptic membrane’s active zone as it does for SVs. The findings are of interest not only because they reveal generality in AZM function and in the construction of different vesicle types at frog NMJs, but also because they may be useful for determining whether the orientation of a predetermined fusion domain on a vesicle membrane for contact with the presynaptic membrane provides advantages for the effectiveness of the vesicle content released into the synaptic cleft.

## Materials and Methods

### Ethics Statement

The animal experimentation described here was approved by Stanford University’s (Protocol Number 10505) and Texas A&M University’s (AUP Number 2011-18) administrative panels on laboratory animal care (IACUC), which oversees the use of animals according to United States federal regulations.

### Muscle Preparation and Sections

The DCV data was gathered from tissue sections cut from cutaneous pectoris muscles of adult *Rana pipiens* (about 5 cm nose-rump length) variously prepared for previously published studies on SVs (Szule et al., 2012; Jung et al., 2016). Much of the SV data we show here for comparison with that from DCVs first appeared in those publications. Thus, details of our methodology are readily available elsewhere, and we refer to such accounts, where appropriate, in “Results” section. Here, we briefly summarize the protocols used in preparing samples used for our observations on the DCVs, which we are offering for the first time.

#### Resting Neuromuscular Junctions

The frogs were anesthetized in 1% phosphate buffered MS-222 (pH 7.2; Sigma Chemical, St Louis, MO, United States) and pithed. The cutaneous pectoris muscles were exposed and, under a dissection microscope, 0.8% glutaraldehyde (Ted Pella, Inc., Redding, CA, United States) in Millonig’s phosphate buffer (220 mOsM, pH 7.2) was injected beneath them and dripped onto their superficial surface several times over 40 min. Some muscles were treated with 10 mg of tetrodotoxin (Sigma-Aldrich) per ml frog Ringer’s solution (115 mM NaCl, 2 mM KCl, 2.2 mM CaCl_2_, 1 mM NaHPO_4_⋅H_2_O; 220–230 mOsM; pH 7.2) for 5 min before fixation to inhibit any glutaraldehyde evoked nerve/muscle impulses; we observed no structural differences in the active zones from treated and untreated muscles by electron tomography. The muscles were removed, pinned flat in a Sylgard 184 (Dow Corning, Midland, MI, United States) coated petri dish containing phosphate buffer (220 mOsM, pH 7.2) and placed on a shaker for 30 min. The muscles were then refixed and stained for 1 h in 1% osmium tetroxide in phosphate buffer (220 mOsM total; pH 7.2), washed 1 h in H_2_O, stained 1 h in saturated aqueous uranyl acetate, dehydrated in increasing concentrations of ethanol and embedded flat in a wafer of Eponate 12 (Ted Pella, Inc., Redding, CA, United States) less than 1 mm thick. Regions of the muscles containing NMJs were identified in the wafers at ×400 magnification with a light microscope, and blocks from these regions were cut out and mounted for sectioning. The sections varied from 50 nm to 150 nm in thickness. They were stained with uranyl acetate in methanol and with aqueous lead citrate.

#### Activated Neuromuscular Junctions

The muscles along with a 5–10 mm stretch of their nerve were removed and pinned out in a Petri dish containing Ringer’s solution. In order to fix the neuromuscular junctions during impulse-evoked synaptic activity, the cut end of the nerve was first drawn into a suction electrode and synaptic activity was tested by passing single current pulses trough the suction electrode while monitoring muscle contractions under a dissection microscope. Fixing muscles during contractions can lead to difficulties in analyzing structural relationships in the electron tomography data. To block muscle contractions during fixation, we replaced the Ringer’s solution with Ringer’s solution containing 10^-5^ g/ml (+)-tubocurarine chloride hydrate (Sigma-Aldrich, Inc., St. Louis, MO, United States) for 5 min. The tubocurarine-containing Ringer’s solution was then replaced with Ringer’s solution containing 0.8% glutaraldehyde (220 mOsM total; pH 7.2), while simultaneously stimulating the nerve with 9–15 μA of current delivered at a frequency of 10 Hz for a duration of 2 min; previous experiments without prior treatment with tubocurarine showed that the neuromuscular junctions were fixed by 2 min. The muscles remained in fixative for an additional 40 min after stimulation had ceased and further processed/sectioned for electron tomography, as described above.

### Data Collection

Tilt-image datasets were collected using either a FEI TF30 Polara electron microscope (FEI Company, Hillsboro, OR, United States) equipped with a 2048x2048 Tietz TemCam-F224HD CCD (Tietz Video and Imaging Processing Systems GmbH, Gauting, Germany) or an FEI Tecnai G2 F20 electron microscope (FEI Company, Hillsboro, OR, United States) equipped with a 2048x2048 Gatan Tridiem GIF-CCD (Gatan, Inc., Pleasanton, CA, United States). The stage on each microscope was cooled to liquid nitrogen temperature to reduce specimen shrinkage. Datasets consisted of images taken at 1° tilt intervals to ±60° or ±70° either along a single tilt axis or along each of two orthogonal tilt axes. They were collected at magnifications ranging from 53,000× to 125,000×.

The images were automatically aligned using 5–10 nm gold colloid (British Biocell International, Cardiff, United Kingdom) fiducial markers deposited on one side of the sections. The average alignment error of datasets in this study was 1.7 ± 1.1 pixels (0.66 ± 0.51 nm). The 3D reconstructions were made by a weighted back-projection method. The software package, EM3D^1^ (Ress et al., 1999, 2004) was used for all image alignments and dataset reconstructions.

### Virtual Slices, Segmentation, and Rendering Surface Models

Virtual slices through the reconstructed tissue sections were 1 voxel thick. Depending on the magnification of the images in a dataset, the virtual slice thickness represented 0.35–1.16 nm of the tissue section’s thickness. When necessary, the angular orientation of the slice plane was adjusted to maximize contrast boundary discrimination of the structures under study.

Structures were segmented from the reconstructions by using a combination of manual and semi-automatic methods in EM3D to define individual volumes-of-interest (VOIs; Ress et al., 2004). For the presynaptic membrane and vesicle membranes, which were heavily stained and had a simple geometry, a semi-automatic scheme was used and manually adjusted as necessary. For structures that had a complex geometry and light to moderate stain such as AZM macromolecules, non-AZM macromolecules and macromolecules in the vesicle lumen, VOIs were defined by manually marking a closed path on the series of slices in which they were included. The VOIs were slightly larger than the structures that they enclosed to allow accurate and complete isodensity-surface calculations for the surface models.

We used EM3D to render a surface model for each VOI. For most structures, the rendering was done using a gray scale value that maximized the area weighted sum of the gray-scale spatial gradient enclosed in the VOI and minimized the mean spatial uncertainty averaged across the whole area of the model (Ress et al., 2003, 2004). Surface models generated in this way had a spatial resolution equal to the resolution of the reconstructed volumes. The macromolecules of the dense core in the lumen of DCVs were segmented and rendered using an automatic optimization method (Jung and Szule, 2017).

### Measurements

#### Connection Sites

To mark the connection sites of AZM macromolecules, non-AZM macromolecules and nubs on the DCV membrane, a 3 voxel thick region of the macromolecule at its intersection with the membrane was segmented and 3D surface model of the connection site was generated as described for SVs in Harlow et al. (2001, 2013), Szule et al. (2012), and Jung et al. (2016).

To compare the relative positions of connection sites of AZM and non-AZM macromolecules on the cytosolic surface of a vesicle membrane to the nub connection sites on the luminal surface of the membrane, we converted the 3D spherical plots of the connection sites to 2D Robinson projections (Robinson, 1974). The positions of the spatial coordinates (*x*, *y*, *z*) of the centroids of connection sites were first plotted onto an idealized sphere using IDL software. The 3D plots of the centroids on the idealized sphere were then warped and expressed on a 2D Robinson projection using the MAP_SET procedure of IDL 7.0 (Harlow et al., 2013).

The proximity tool in EM3D (Ress et al., 2004) was used to measure the direct distances between the centroids of each connection site of the different classes of AZM and non-AZM macromolecules to the closest point on the presynaptic membrane, and the measurements were each normalized by subtracting the average position of the rib connection sites per vesicle. Here, the distance of the centroid of each connection site to the presynaptic membrane was determined as the average distance of all of its voxels to the nearest voxel of the presynaptic membrane.

#### Surface Area

The total surface area of each DCV (*S_DCV_*) was calculated according the formula for measuring the surface area of a sphere:

$$S_{DCV}=\pi d_{v}^{2}$$

For DCVs completely included in the reconstruction, the diameter (*d_v_*) was measured to the cytosolic surfaces of the membrane in all three cardinal planes and averaged. For DCVs that were not completely included in the reconstruction, the diameter all three cardinal planes was determined from the curvature of the membrane, and averaged.

The surface area of the main AZM binding domain (AZM-BD) and the fusion domain (FD) were calculated as caps of the sphere based on the diameter of the domain (*d_Domain_*: *d_AZM-BD_* or *d_FD_*) and the diameter of the vesicle (*d_V_*) using the following formula:

$$S_{Domain}=\frac{\pi }{2}d_{v}\left(d_{v}-\sqrt{d_{v}^{2}-d_{Domain}^{2}}\right)$$

### Figure Layouts

Figure layouts were prepared using Adobe Photoshop CS3 (Adobe Systems, San Jose, CA, United States). The gray-scale levels and curves for were adjusted slightly in Photoshop to optimize the fidelity of the electron micrograph images for publication and reproduction.

The RGB color values for the surface models are as follows: presynaptic membrane (200, 200, 225); vesicle membranes (125, 125, 255); ribs (255, 197, 31); beams (125, 75, 25); pegs (255, 175, 0); pins (200, 100, 25); steps (255, 255, 255); spars (255, 0, 0); masts (0, 100, 0); booms (100, 0, 150); topmasts (100, 150, 0); non-AZM macromolecules (200, 200, 200).

## Results

Because of the thinness of the sections required for best spatial resolution (50–150 nm thick), some active zones at which there were DCVs included only portions of DCVs. Nevertheless, the reconstructions of the active zones that included entire DCVs together with those that included various fractions of DCVs provided sufficient information for quantitative comparison of the structural relationships of DCVs to the relationships previously reported for SVs. To facilitate such comparison, we show here not only images from this study on DCVs, but also selected images from our previous electron tomography studies on SVs.

**FIGURE 1:**
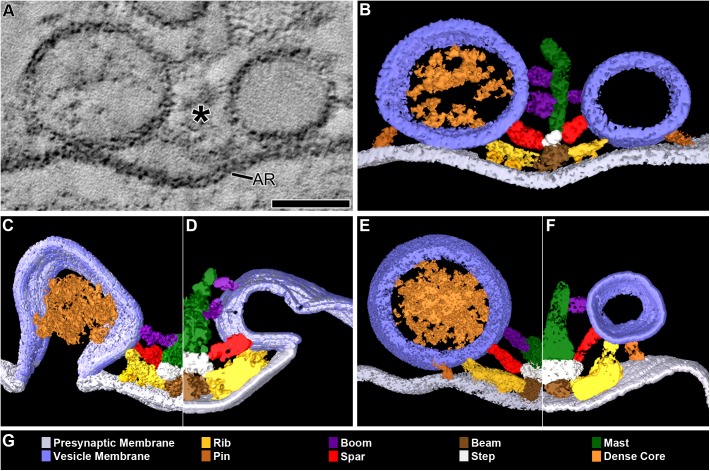
DCVs dock on and fuse with the presynaptic membrane alongside SVs at active zones. **(A)** A 3 nm thick virtual slice from a reconstructed 110 nm thick tissue section cut in the transverse plane of an active zone at a resting neuromuscular junction. The active zone ridge (AR) of the presynaptic membrane and main body of AZM (^∗^), which extends from the ridge into the cytosol are flanked by a DCV (left) and SV (right). Scale bar = 50 nm. **(B)** Surface models of structures in **(A)** generated from 42 serial virtual slices (18 nm total) through the reconstructed volume. Both vesicle types are in contact with the presynaptic membrane and are connected to AZM macromolecules. Thus, they are docked. The AZM macromolecules connected to the vesicles include ribs, spars, and booms in the main body of the AZM and pins away from the main body. The ribs, spars, and booms are also connected to a beam, step, and mast, respectively, at the midline of the main body. Pegs link the ribs to the presynaptic membrane. Pins link the vesicles directly to the presynaptic membrane. **(C–F)** Surface models generated as in **(B)** from active zones fixed during repetitive synaptic transmission. A former docked DCV and SV that had fused with the presynaptic membrane are shown in **(C,D)** respectively. Each is at a stage of flattening into the presynaptic membrane where the AZM macromolecules connected to docked vesicles are still attached. A docking DCV and SV are shown in **(E,F)**, respectively. Both are within 15 nm of the presynaptic membrane and are connected to the same assortment of AZM macromolecules as docked vesicles **(B)**. **(G)** Color code for components in **(B–F)**. All images are from muscles fixed with aqueous glutaraldehyde and stained with aqueous solutions of osmium tetroxide and uranyl acetate. Non-AZM macromolecules connected to the vesicle were not included in the surface models. **(D,F)** Taken from Szule et al. (2012).

The active zones of the presynaptic membrane at frog neuromuscular junctions are ∼150 nm wide and can be more than 1 μm long (Couteaux and Pecot-Dechavassine, 1970; Heuser et al., 1974). The tissue sections used for this study were cut near the active zones’ transverse plane (Szule et al., 2012). When viewed in this plane, the shallow evagination of the presynaptic membrane, the active zone ridge, that extends the length of the active zone is seen in cross section (**Figure 1**). The ridge is flanked on each side by one or, depending on the thickness of the section, a row of two or more vesicles a few nm apart that extend alongside the ridge in the section’s depth axis (**Figures 1A,B**). The main body of AZM, which stretches the full length of the active zone, is positioned between the rows of vesicles. It is attached to the presynaptic membrane along the slopes of the ridge and projects 50 nm to 75 nm vertical to the membrane into the cytosol (Harlow et al., 2001; Szule et al., 2012).

### Docking on and Fusion With the Presynaptic Membrane at Active Zones

In 77 randomly selected reconstructions of active zones from five muscles fixed at rest there were 351 vesicles flanking the active zone ridge and AZM. Six were DCVs, and 345 were SVs. As detailed below, all were connected to AZM and non-AZM macromolecules. The DCVs in 3D surface models were ellipsoidal as are SVs (Jung et al., 2016). The averaged diameter of each ranged from 78 nm to 92 nm (84 nm average), which falls within the range of diameters reported for DCVs in the terminal’s general vesicle population, as measured by conventional electron microscopy on tissue sections (Pecot-Dechavassine and Brouard, 1997). From electron tomography data collected for a previous study on 101 docked SVs at similarly prepared frog neuromuscular junctions, the averaged diameter for each SV ranged from 48 to 68 nm (56 nm average) (Jung et al., 2016).

Four of the six DCVs were docked on the presynaptic membrane (**Figures 1A,B**). Specifically, in virtual slices there was no discernable gap between the DCV membrane and the presynaptic membrane at their closest points of apposition, as shown for docked SVs (Jung et al., 2016). For the two other DCVs there was a narrow gap (<15 nm) between the vesicle membrane and the presynaptic membrane at their closest points of apposition. Three hundred thirty-three SVs were docked on the presynaptic membrane; 12 were <15 nm from it. Based on our previous study on the sequence of steps in the docking of SVs (Szule et al., 2012), both the DCVs and SVs <15 nm from the presynaptic membrane were likely to be at the penultimate docking step (**Figures 1E,F**). Thus, we refer to such vesicles below as “docking” DCVs and SVs.

Previous studies have shown that in muscles fixed with aldehydes during repetitive impulse-evoked synaptic transmission there are not only docked and docking SVs flanking the AZM, but also former docked SVs captured by the fixative in different stages of flattening into the presynaptic membrane after their membrane had fused with it (Heuser et al., 1979; Szule et al., 2012). In early stages, profiles of the fused SV membrane are somewhat Ω-shaped, and the membrane remains connected to the AZM macromolecules (**Figure 1D**; Szule et al., 2012). Out of 62 reconstructions of active zones from two muscles in which the nerve innervating them had been electrically stimulated at 10 Hz for 2 min beginning with the application of glutaraldehyde to the muscles, we found four DCVs alongside AZM. The averaged diameter of each ranged from 64 to 115 nm (88 nm average). All were connected to AZM macromolecules. Of these, two were docked on the presynaptic membrane and two were docking. Also, alongside and connected to the AZM macromolecules was an Ω-shaped profile containing a dense core, which we interpret as a former DCV that had fused with the presynaptic membrane (**Figure 1C**).

### Number and Distribution of AZM and Non-AZM Connection Sites

**FIGURE 2:**
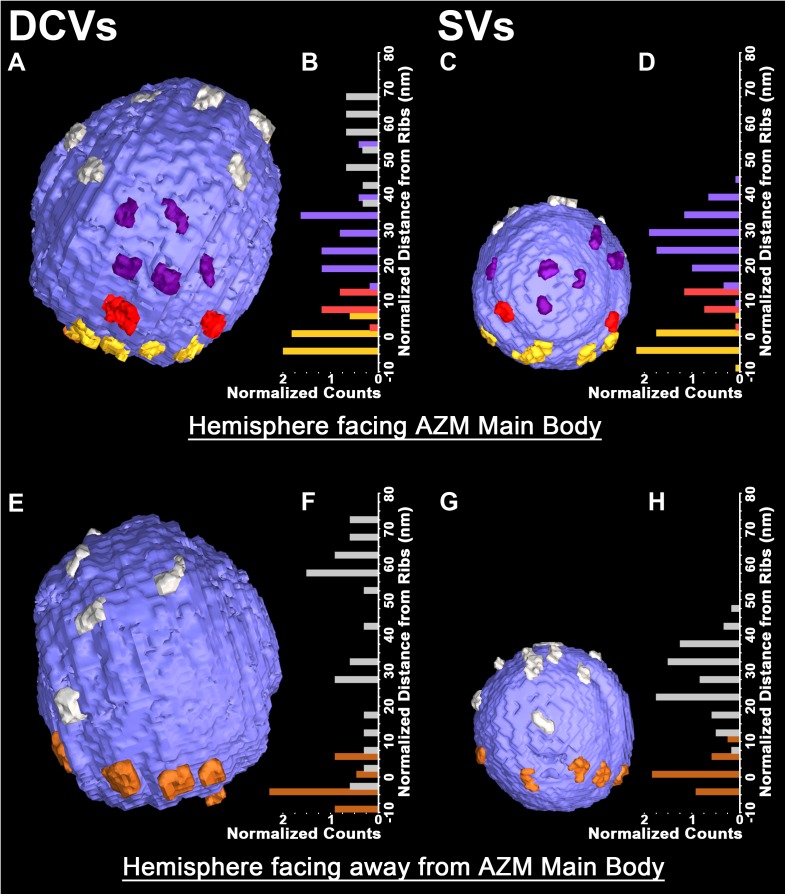
Similarities in the distribution of the connection sites of AZM and non-AZM macromolecules on the membrane of docked DCVs and SVs. **(A,C)** Hemisphere of a docked DCV and SV facing the main body of AZM. The connection sites of ribs, spars, and booms (color coded as in **Figure 1**) are confined to similar sized area on the vesicle membrane, the main AZM binding domain. Connection sites of non-AZM macromolecules (pewter) occur beyond this domain. **(B,D)** For each of the 5 DCVs and 12 SVs in our samples that included the entire hemisphere facing the main body of AZM, the connection sites of ribs, spars, and booms, occupy class specific bands nearly parallel to the presynaptic membrane; the ribs are closest to the presynaptic membrane and booms are farthest from it, as seen in **Figure 1**. **(E,G)** Hemisphere of the docked DCV and SVs in **(A)** and **(B)** facing away from the main body of AZM showing the connection sites of pins (color coded as in **Figure 1**) and non-AZM macromolecules. **(F,H)** For each of the DCVs and SVs in our samples that included the entire hemisphere facing away from the main body of AZM (see **Table 1** for *n* values) the connection sites of the pins were near the presynaptic membrane while those of the non-AZM macromolecules were broadly distributed and further from it. All images are from muscles fixed with an aqueous solution of glutaraldehyde and stained with aqueous solutions of osmium tetroxide and uranyl acetate. **(D,H)** Taken from Szule et al. (2012) and Harlow et al. (2013).

**FIGURE 3:**
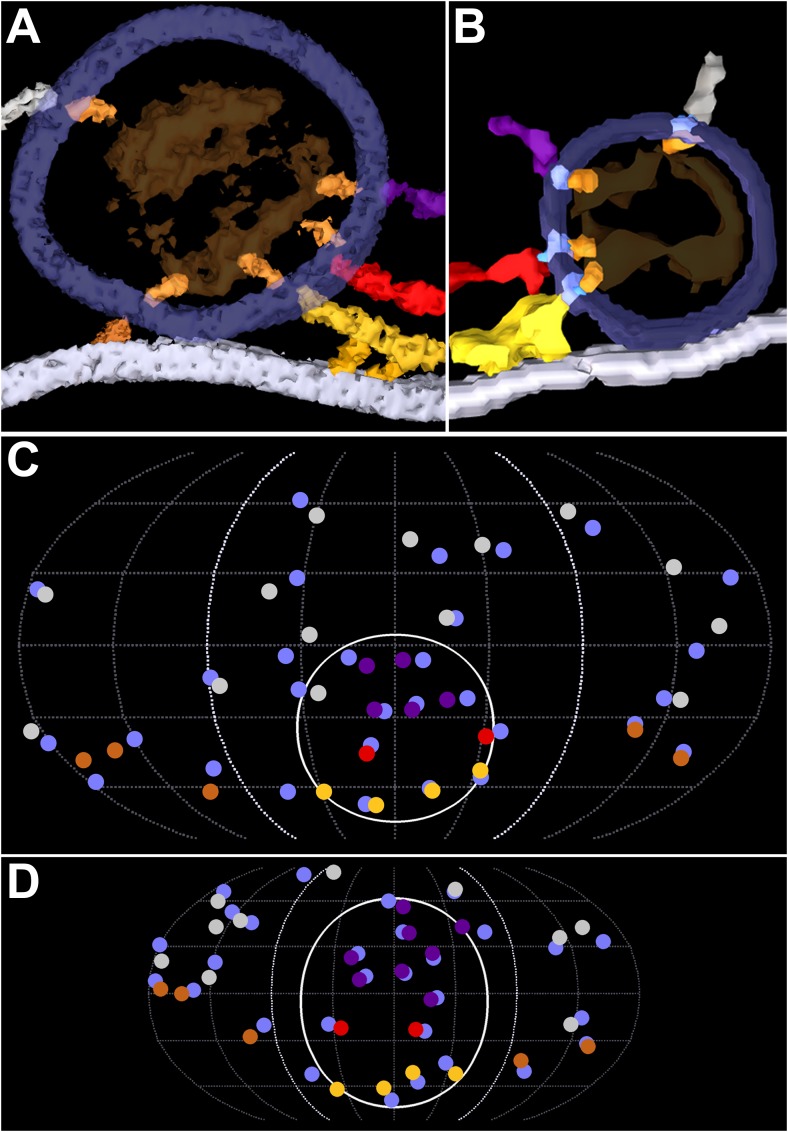
Similarity in the pairing of nub connection sites with AZM and non-AZM macromolecule connection sites on the membrane of DCVs and SVs. **(A,B)** Nubs (bright orange) in 10 nm thick surface models link the dense core/luminal assembly (ghosted orange) of macromolecules in a DCV and the luminal assembly of macromolecules in a SV to the luminal surface of the vesicle membrane (ghosted dark blue). The nubs are aligned with the connection sites of ribs, pins, spars, booms, and non-AZM macromolecules (color coded as in **Figures 1**, **2**). **(C,D)** Robinson maps showing all connection sites of nubs (blue) on the luminal surface of the membrane of a docked DCV **(C)** and a docked SV **(D)**. The nubs are opposite or slightly offset from the connection sites of the ribs, pins, spars, booms, and non-AZM macromolecules (color coded as in **Figures 1**, **2**). The hemisphere of the vesicles facing the main body of the AZM lies between the bold dotted longitudinal lines. The solid white line encircles the main AZM binding domain. Data for DCVs are from muscles fixed with an aqueous solution of glutaraldehyde and stained with aqueous solutions of osmium tetroxide and uranyl acetate. Data for SVs are from muscles fixed by rapid freezing and stained by freeze-substitution of osmium tetroxide and uranyl acetate in acetone, a method which reveals not only the pairing of nubs with the connection sites of AZM and non-AZM macromolecules on the SV membrane, but also transmembrane macromolecules shown in **(B)** (pale blue) that link them together. **(B,D)** Taken from Harlow et al. (2013).

The membrane of each of the 10 docked or docking DCVs at active zones from resting and active NMJs was connected to one end of the same four classes of AZM macromolecules that connect to the membrane of docked and docking SVs (**Figures 1A,B,E,F**). Three of the classes, so-called ribs, spars and booms, were components of the main body of AZM. Each class terminated on the DCV hemisphere that faced the main body as it does on SVs, and each class was connected at its opposite end to a specific class of macromolecules at the main body’s midline (**Figure 1**). The ribs were also connected, along their length, to two pegs (**Figure 1B**), which linked them to macromolecules in the presynaptic membrane thought to include Ca^2+^ and K^+^ channels as are the ribs attached to SVs (Harlow et al., 2001; Jung et al., 2016). The membrane of the former docked DCV that had fused with and had begun flattening into the presynaptic membrane during impulse activity was also connected to ribs, spars and booms as has been observed for fused SVs in the early stage of flattening (**Figures 1C,D**). The hemisphere facing the main body of AZM was fully included in the reconstructions of three docked DCVs, one docking DCV, and the DCV that had fused with the presynaptic membrane. On average, the five DCV membranes were connected to four ribs, two spars, and six booms, which are not significantly different from the average numbers of ribs, spars, and booms connected to the membrane of docked and docking SVs (**Table 1**). The connection sites of each class of AZM macromolecule on the five DCV membranes were situated in distinct but overlapping bands that paralleled the plane of the presynaptic membrane, as they are on the membrane of docked SVs, with the connection sites of ribs nearest to and those of booms farthest from the plane of the presynaptic membrane (**Figure 2**). Moreover, the average distances between the connection sites of ribs, spars and booms on the membrane of the DCVs were not different from the average distances between the connection sites of ribs, spars, and booms on the membrane of docked SVs (**Figure 2** and **Table 1**). The bands of connection sites of ribs, spars, and booms were confined to a circular area, the main AZM binding domain, on the DCV membrane, as they are on the SV membrane (**Figures 2**, **3**). Its diameter was on average 40 nm, which is not significantly different from the average diameter of the main AZM binding domain on SVs (**Figure 3** and **Table 1**). As specified below, the average area of main AZM binding domain on both DCVs and SVs covers only a small fraction of the total DCV and SV surface area, although the fraction is somewhat greater on SVs because of their smaller surface area (**Table 1**).

**Table 1:** Comparison of the structure and relationships of dense core vesicles to those of synaptic vesicles.

	Dense core vesicles		Synaptic vesicles				
	Mean ± SD	*n*	Mean ± SD	*n*	Reference	*p*-Value	Statistic Test
# of rib connections per vesicle	4.4 ± 0.54	5	3.9 ± 0.6	20	Szule et al., 2012 Harlow et al., 2013	0.08	Mann–Whitney
			4.2 ± 0.4	12		0.35	
# of spar connections per vesicle	2.2 ± 0.45	5	2.2 ± 0.5	20	Szule et al., 2012 Harlow et al., 2013	0.99	Mann–Whitney
			2.0 ± 0.4	12		0.43	
# of boom connections per vesicle	6.0 ± 0.71	5	5.0 ± 2.0	20	Szule et al., 2012 Harlow et al., 2013	0.24	Mann–Whitney
			7.0 ± 0.9	12		0.051	
# of pin connections per vesicle	5.0 ± 1.4	4	4.1 ± 0.7	12	Szule et al., 2012 Harlow et al., 2013	0.16	Mann–Whitney
			3.9 ± 1.3	12		0.15	
# of non-AZM connections per vesicle	12.0 ± 2.6	3	8.5 ± 1.8	10	Harlow et al., 2013	0.04^∗^	Mann–Whitney
Rib-vesicle distance to PM (nm)	10.5 ± 2.8	4	7.7 ± 3.3	20	Szule et al., 2012 Jung et al., 2016	0.13	Mann–Whitney
			12.1 ± 2.5	101		0.22	
Rib-vesicle distance to spar-vesicle (nm)	8.7 ± 2.7	5	10.3 ± 4.4	20	Szule et al., 2012	0.32	Mann–Whitney
Rib-vesicle Distance to boom-vesicle (nm)	27.0 ± 5.3	5	24.2 ± 6.6	20	Szule et al., 2012	0.23	Mann–Whitney
Total surface area of vesicle (nm^2^)	27950 ± 4809	3	8298 ± 1113	12	Harlow et al., 2013	0.012^∗^	Mann–Whitney
AZM binding domain-diameter (nm)	40.9 ± 9.3	5	34.9 ± 4.2	12	Harlow et al., 2013	0.10	Mann–Whitney
AZM binding domain-area (nm^2^)	1508 ± 818	5	1135 ± 312	12	Harlow et al., 2013	0.43	Mann–Whitney
% surface area of AZM binding domain	6.0 ± 3.4	3	13.7 ± 3.6	12	Harlow et al., 2013	0.017^∗^	Mann–Whitney
Vesicle fusion domain area (nm^2^)	2182 ± 1477	4	1426 ± 279	12	Szule et al., 2012	0.43	Mann–Whitney
% surface area of fusion domain	11.1 ± 7.3	3	17.2 ± 3.3	12	Szule et al., 2012	0.22	Mann–Whitney
Vesicle-PM contact area (nm^2^)	310 ± 180	6	330 ± 150	101	Jung et al., 2016	0.71	Mann–Whitney

*SD, standard deviation; PM, presynaptic membrane; ^∗^ indicates that the p-value < 0.05.*

**FIGURE 4:**
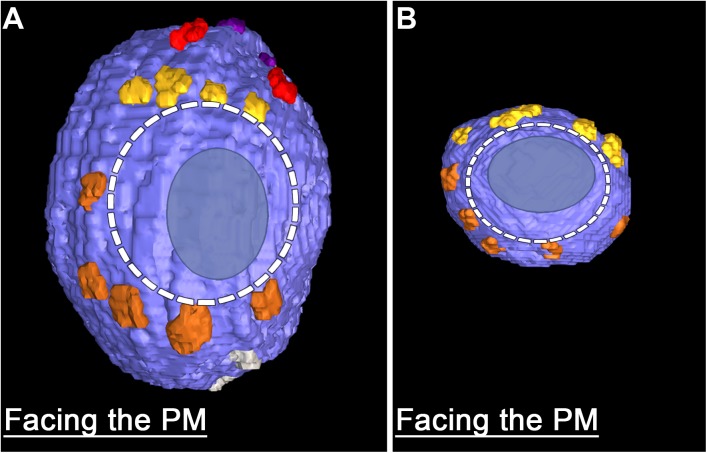
Similarities in the fusion domains of DCVs and SVs. Hemisphere of a docked DCV **(A)** and a docked SV **(B)** also shown in **Figure 2**, facing the presynaptic membrane. Ribs and pins surround both vesicles’ fusion domain (dashed line). The vesicle membrane-presynaptic membrane contact area (blue ovoid patch) occupies only a portion of the fusion domain. Images are from muscles fixed with an aqueous solution of glutaraldehyde and stained with aqueous solutions of osmium tetroxide and uranyl acetate. **(B)** Taken from Szule et al. (2012).

The fourth class of AZM macromolecules connected to the 10 docked and undocked DCVs at active zones fixed at rest or during impulse activity were pins (**Figures 1–4** and **Table 1**). They linked the membrane of DCVs directly to the presynaptic membrane as they do for docked and docking SVs. The length of those connected to DCVs ranged from 5 to 20 nm as does the length of those connected to docked and docking SVs (Szule et al., 2012; Jung et al., 2016). Moreover, an average of five pins was connected to the four docked and docking DCVs in which the hemisphere facing the presynaptic membrane was fully included in the reconstructions. This is not different from the average number of pins connected to docked and docking SVs (**Table 1**). As for SVs, the connection sites of the pins on the DCVs were primarily on the hemisphere facing away from the main body of AZM, and, together with the connection sites of ribs, they encircled the vesicle membrane’s fusion domain (**Figure 4**). The average area of the fusion domain for the four DCVs was 2,200 nm^2^ and on average covered 9% of the surface area, which is similar to the average fraction of SV surface area covered by the fusion domain (**Table 1**). The entire DCV membrane-presynaptic membrane contact area within the fusion domain was evident for each of the six docked DCVs in our sample; the contact areas ranged from 70 to 600 nm^2^, which is similar to the range of 46–630 nm^2^ for a much larger sample (Jung et al., 2016) of SV membrane-presynaptic membrane contact areas (**Table 1**).

**FIGURE 5:**
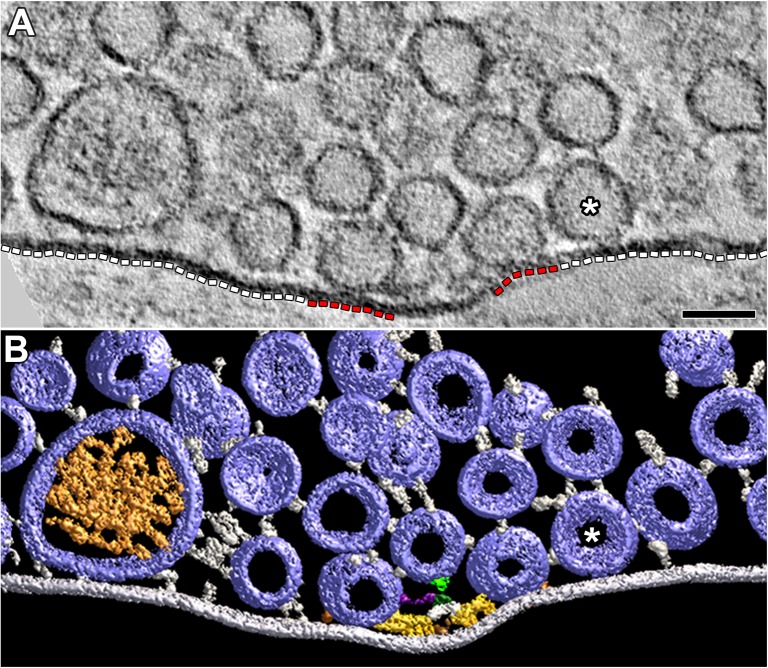
Vesicles and their macromolecular connections at and near the extra-active zone region of the presynaptic membrane. **(A)** 4 nm thick virtual slice showing some SVs and one DCV near the extra-active zone region (white dashed lines) of the presynaptic membrane. One SV (^∗^) is in contact with this region of membrane. Two SVs are in contact with the active zone portions of the presynaptic membrane (red dashed lines). Scale bar = 50 nm. **(B)** Surface models (color coded as in **Figures 1**, **3**) generated from 15 serial virtual slices [the same SV in contact with the presynaptic membrane in **(A)** is also indicated by (^∗^)] reveal many more SVs near the extra-active zone region of the presynaptic membrane than evident in **(A)**. Non-AZM macromolecules connected to the cytosolic surface of these vesicles link the vesicles to the presynaptic membrane and to other vesicles or they appear to end blindly in the cytosol. The docked SVs are also linked to components of the AZM. The mast is connected to an undocked vesicle by a topmast (light green) as described and discussed in Szule et al. (2012); we also noted topmasts extending from other masts shown in this study connected to SVs and DCVs, but we did not include them in the other surface models presented here. The muscle was fixed with an aqueous solution of glutaraldehyde and stained with aqueous solutions of osmium tetroxide and uranyl acetate.

In addition to AZM macromolecules, the 10 docked or docking DCVs at active zones fixed at rest and during impulse activity, as well as the former docked DCV that had fused with the presynaptic membrane, were connected to elongate non-AZM macromolecules (e.g., **Figures 2**, **3**). The three docked DCVs that were fully included in the reconstructions had an average of 12 connection sites of the non-AZM macromolecules, which is significantly greater than the average of nine connection sites of non-AZM macromolecules on docked and docking SVs (**Figures 2**, **3** and **Table 1**). The 12 connection sites of the non-AZM macromolecules had a broad distribution over the DCV surface beyond the main AZM binding domain and the fusion domain as do the non-AZM connection sites on SVs (**Figures 2**, **3**). Like the non-AZM macromolecules connected to SVs, those connected to DCVs linked the DCVs to vesicles and other organelles beyond the active zone (**Figure 5**), and they were similar in appearance to macromolecules that connect undocked vesicles to each other and other organelles throughout the terminal (Llinas et al., 1985; Landis et al., 1988; Hirokawa et al., 1989; Feng et al., 2002; Szule et al., 2012; Cole et al., 2016).

A summary of the overall distribution of macromolecule connection sites on the cytosolic surfaces of DCVs and SVs is as follows. For DCVs none was in the fusion domain (9% of the total surface area), 40% were within the main AZM binding domain (6% of the total surface area), and 60% were broadly distributed over the region beyond these two domains (85% of the total surface area). For SVs none is in the fusion domain (17% of the total surface area), 48% are within the main AZM binding domain (14% of the total surface area), and 52% are broadly distributed over the region beyond these two domains (69% of the total surface area). Thus, there was on the cytosolic surface of docked DCVs a stereotypic non-uniform distribution of macromolecule connection sites with the highest concentration in the main AZM binding domain, similar to that on SVs (**Figures 2–4**).

### Dense Core Macromolecules and the Pairing of Nub Connection Sites With AZM and Non-AZM Connection Sites

**FIGURE 6:**
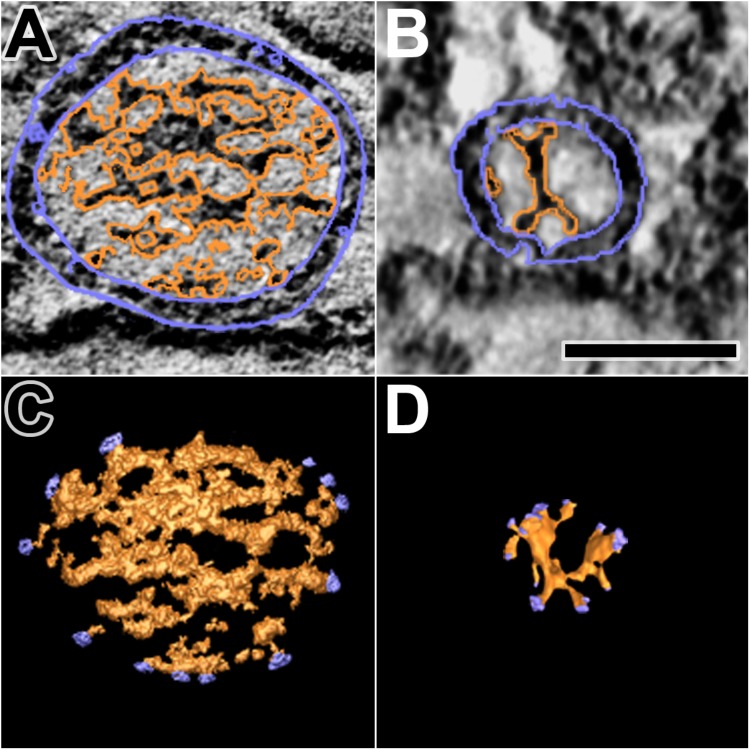
Luminal assemblies of macromolecules in DCVs and SVs. **(A)** A 3 nm thick virtual slice of a docked DCV, and **(B)** a 1 nm thick virtual slice of a docked SV showing the luminal and cytosolic surfaces of the vesicle membrane (outlined in blue) and the luminal assembly of macromolecules (outlined in orange). **(C)** A surface model from 10 virtual slices including the one in **(A)**. **(D)** A surface model from 35 virtual slices including the one in **(B)**. The sites where nubs link the luminal assemblies to the vesicle membrane are blue. The luminal assembly in the DCV comprises the dense core. The DCV is from a muscle fixed with an aqueous solution of glutaraldehyde and stained with aqueous solutions of osmium tetroxide and uranyl acetate. The SV is from a muscle fixed by rapid freezing and stained by freeze-substitution of osmium tetroxide and uranyl acetate in acetone. **(B,D)** Taken from Harlow et al. (2013). Scale bar = 50 nm.

The dense core in each of the 10 docked or docking DCVs at active zones contained an interconnected assembly of irregular elongate macromolecules. This assembly differed from the luminal assembly of macromolecules previously described for SVs at frog neuromuscular junctions (Harlow et al., 2013) in that it had a greater abundance of macromolecules and the macromolecules had a greater packing density as determined by eye (**Figure 6**). It also differed in its stainability; the luminal assembly of macromolecules in SVs is not evident in preparations stained by immersion in aqueous osmium tetroxide and uranyl acetate at room temperature, as used for this study of DCVs, but rather by freeze-substitution of osmium tetroxide and uranyl acetate in acetone (**Figure 6**) (Harlow et al., 2013). Nevertheless, nubs linked the assembly of macromolecules in DCVs to the luminal surface of the DCV membrane just as nubs link the assembly of macromolecules in SVs to the luminal surface of the SV membrane. For the three DCVs entirely included in the tissue sections, there were 25 (24.7 ± 0.58 SD) nub connection sites on the luminal surface of the vesicle membrane, which was not significantly different from the total number of 29 (29.0 ± 5.3 SD) connection sites of AZM and non-AZM macromolecules on the outer surface of the membrane (*p* = 0.16, Mann–Whitney test). As for docked SVs (Harlow et al., 2013), there was a stereotypic non-uniform arrangement of nub connections sites on the DCV membrane (**Figure 3**); although there was a broad distribution over the membrane surface, there were no nub connection sites in the fusion domain, and the greatest concentration of connection sites was in the membrane’s main AZM binding domain. Moreover, as for SVs (Harlow et al., 2013), in nearly every case (90%) the connection site of a nub was paired with (i.e., opposite or slightly offset from) the connection site of an AZM or non-AZM macromolecule (**Figure 3**), and in nearly every case (89%) the connection site of an AZM or non-AZM macromolecule was paired with a nub. The nubs and AZM/non-AZM macromolecules that were not paired may have been due to inadequate staining of one member of a pair.

### Vesicles in Contact With the Region of Presynaptic Membrane Beyond the Active Zone

The portion of the axon terminal’s presynaptic membrane directly opposite the muscle fiber membrane in each of our reconstructions extended several hundred nanometers beyond the active zone. The adjacent cytosol contained numerous SVs and a few DCVs that were a part of the vesicle cloud centered on the active zone (**Figure 5**). For the extra-active zone regions of presynaptic membrane in the 77 reconstructions from resting axon terminals, the membrane of 64 SVs and 1 DCV (not shown) was in contact with the presynaptic membrane. For the active zone regions of presynaptic membrane, the membrane of 333 SVs and 4 DCVs were in contact with the presynaptic membrane. To compare the concentration of DCVs in contact with the portion of the active zone and extra-active zone regions of the presynaptic membrane, we divided the number of vesicles by the area of each region. The active zone regions were in the shadow of the vesicles flanking the main body of AZM (**Figure 5A**). The extra-active zone regions were beyond the active zone region (**Figure 5A**). Based on the total area of the active zone region of presynaptic membrane (907,000 μm^2^) and the total area of the extra-active zone region of presynaptic membrane (3,282,000 μm^2^), we found that the concentrations of SVs and of the DCVs in contact with the active zone region were, respectively, ∼20-fold and ∼15-fold greater than the concentrations of those in contact with the extra-active zone region.

Several elongate macromolecules extended from the membrane of the DCV and the SVs to the extra-active zone region of the presynaptic membrane (**Figure 5**). None of the macromolecules was part of a dense organized network such as that constituting the main body of AZM (**Figure 1**). Rather, they were apart from one another and indistinguishable from the elongate macromolecules that link vesicles throughout the terminal to each other and to other organelles (Llinas et al., 1985; Landis et al., 1988; Hirokawa et al., 1989; Feng et al., 2002; Szule et al., 2012; Cole et al., 2016).

## Discussion

**FIGURE 7:**
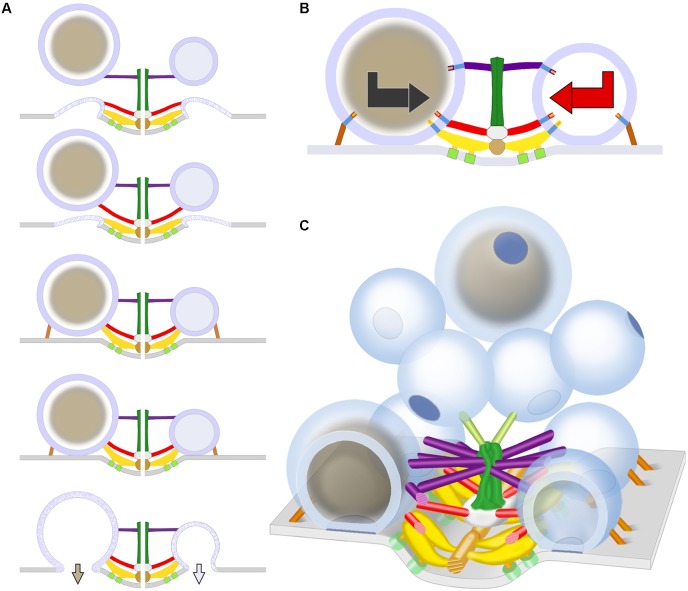
Schematic of predicted steps in AZM directed orientation, docking, and fusion of DCVs alongside SVs at active zones of the presynaptic membrane. **(A)** Docking steps for a DCV (left) based on the similarity of its AZM connections to those of SVs (right; modified from Szule et al., 2015) at the penultimate and ultimate docking steps, and on what is known about the preceding docking steps for SVs. When booms, spars, and, together, ribs and pins (color coded as in **Figure 1**) sequentially disconnect from a previously docked SV that had fused with the presynaptic membrane and is flattening into it, a DCV sequentially connects to the different classes of AZM macromolecules, which brings the fusion domain of the DCV membrane into contact with the presynaptic membrane and holds it there until the arrival of a nerve impulse, which triggers DCV membrane-presynaptic membrane fusion that will be followed by exocytosis of the DCV’s cargo. The docking sites are adjacent to macromolecules in the presynaptic membrane (light green) thought to include Ca^2+^ and K^+^ channels which are involved in the regulation of vesicle fusion during synaptic transmission and are linked by pegs to ribs (Harlow et al., 2001; Szule et al., 2012; Jung et al., 2016). **(B)** The docking of DCVs and SVs relies on specific sets of nubs and their transmembrane macromolecules aligning with and connecting to booms, spars, ribs, and pins (the nubs are color coded according to the class of AZM macromolecule to which they are connected). This results in bidirectional vesicle orientation with respect to the main body of the AZM and presynaptic membrane, as indicated by the bidirectional arrows. Modified from Harlow et al. (2013) to include a DCV. **(C)** As for undocked SVs away from the active zone, the predetermined fusion domain (blue patch) on undocked DCVs away from active zones is not oriented in any particular direction relative to the presynaptic membrane and AZM. When the vesicles are motile, it is the alignment of the predetermined arrangement of nubs and their transmembrane macromolecules in the vesicles’ membrane with AZM macromolecules, which must occur for docking to proceed, that directs the fusion domain toward the presynaptic membrane. Modified from Szule et al. (2015) to include DCVs.

We show that DCVs are distributed at low frequency among the linear arrays of SVs along the presynaptic membrane’s active zones at frog neuromuscular junctions. All were connected to AZM and non-AZM macromolecules as are the SVs. Some were in contact with the presynaptic membrane, at the 2–3 nm spatial resolution provided by our methods, as are docked SVs. Others were <15 nm from the presynaptic membrane as are SVs in the process of docking. Fixing the tissue during repetitive electrically evoked synaptic impulse transmission captured the membrane of a DCV connected to AZM that had fused with the presynaptic membrane and had begun flattening into it as observed for some SVs under the same conditions (Szule et al., 2012). Thus, we conclude that DCVs dock on and fuse with the presynaptic membrane at active zones of neuromuscular junctions during normal synaptic activity as do SVs (schematized in **Figure 7A**), although at a much lower frequency.

The DCVs at active zones in our samples had averaged diameters ranging from 64 to 115 nm, and the general appearance of their dense cores was similar. Others using conventional electron microscopy on tissue sections from frog neuromuscular junctions prepared in a way similar to the one we used, observed DCVs throughout the entire vesicle population (Pecot-Dechavassine and Brouard, 1997). They found a somewhat broader range of diameters (50–150 nm) and a greater variability in the staining contrast of the dense cores than those we observed at and near active zones. Such variabilities led them to suggest that there are several structurally distinct classes of DCVs in the terminals. Thus, the similarity of the dense cores and narrower size range of the DCVs in our samples may mean that only members of a particular class of DCVs dock on and fuse with the presynaptic membrane at active zones. Alternatively, all classes might dock and fuse at active zones, but our sample number was not large enough to include members of each. Nevertheless, from the findings we present here and elsewhere, as discussed below, it would seem that any DCV that docks on and fuses with the presynaptic membrane at active zones must have certain structural features in common with SVs and those DCVs we observed at active zones that enables AZM to direct them to the docking/fusion sites.

We found that DCVs and SVs can come into contact with the extra-active zone region of the presynaptic membrane. Such vesicles were linked to the extra-active zone presynaptic membrane by macromolecules similar in appearance and arrangement to non-AZM macromolecules linking vesicles to each other and to other types of organelles throughout the cytosol, which apparently serve to regulate vesicle movement in general (Llinas et al., 1985). Based on conventional electron microscope images from frog neuromuscular junctions, others have suggested that DCVs may fuse with the axonal plasma membrane opposite the narrow Schwann cell processes that enwrap the axon terminals at intervals along their length (Pecot-Dechavassine and Brouard, 1997). Our samples did not include the Schwann cell enwrapments. Nevertheless, our observation that the concentration of DCVs and SVs in contact with the presynaptic membrane is 15- to 20-fold greater at active zones than in the extra-active zone region indicates that DCVs of the type we observed dock on and fuse with the presynaptic membrane selectively at active zones, as has been long accepted for SVs (Heuser et al., 1979).

Docked and docking DCVs at active zones were connected to the same structurally distinct classes of AZM macromolecules that connect to docked and docking SVs: ribs, pins, spars, and booms (Szule et al., 2012). Moreover, the connection sites of the different classes had the same number and orderly arrangement on the membrane of DCVs as they do on the membrane of SVs; those of the ribs, spars and booms were confined to class specific bands on a small area of the vesicle membrane facing the main body of AZM, the membrane’s main AZM binding domain, while the connections sites of ribs and pins surrounded the vesicle membrane’s fusion domain, which was devoid of any macromolecule connection sites. The connection sites of non-AZM macromolecules on the membrane of DCVs also had a distribution similar to those on the membrane of SVs (Harlow et al., 2013), being broadly distributed over the vesicle surface beyond the main AZM binding domain and fusion domain. They were somewhat more abundant than on SVs, which is perhaps related to the increased size of the DCVs. Nevertheless, there was a stereotypic non-uniform distribution of macromolecule connection sites with the highest concentration in the main AZM binding domain for both DCVs and SVs. The inclusion of docked and docking DCVs among the rows of SVs alongside AZM, the similarity in appearance of the AZM and non-AZM macromolecules connected to docked and docking DCVs to that of the AZM and non-AZM macromolecules connected to SVs, and the similarity in the arrangement of the connection sites on both vesicle types would be expected if the same AZM and non-AZM macromolecules that regulate the docking and fusion of a SV also regulate the docking and fusion of a DCV.

As observed for SVs (Harlow et al., 2013), nearly all connection sites of AZM and non-AZM macromolecules on the cytosolic surface of the DCV membrane were paired with the connection sites of nubs on the luminal surface, and nearly all connection sites of nubs were paired with the connections sites of AZM and non-AZM macromolecules. Our previous studies on SVs using a different staining method than the one used for this study revealed that the nubs are linked to their paired AZM and non-AZM macromolecules by a transmembrane macromolecule (**Figure 7B**) (Harlow et al., 2013). The staining method we used here did not reveal such macromolecules in the membrane of either SVs or DCVs. However, the pairing of the nub connection sites on the luminal surface of the DCV membrane with the AZM and non-AZM connections sites on the cytosolic surface is consistent with the likelihood that the AZM macromolecules are connected to a specific set of nub-linked transmembrane macromolecules in the membrane of DCVs as are AZM macromolecules connected to the membrane of SVs.

The arrangement of the luminal assembly of macromolecules in undocked SVs away from active zones, including the assembly’s nubs and their connection sites on the vesicle membrane, is similar to that for docked SVs. This has been taken to indicate that the arrangement of the connection sites of the AZM and non-AZM macromolecules and the fusion domain on docked SVs is predetermined on the undocked vesicles (Harlow et al., 2013). However, from one undocked SV to another away from active zones there is no particular orientation of this arrangement with respect to AZM and the presynaptic membrane, indicating that such orientation must occur for AZM-directed docking to proceed. It seems likely that the AZM connection sites are predetermined on undocked DCVs as on undocked SVs and that the arrangement of the predetermined connection sites must also undergo orientation before the connections can be made. Accordingly, the sequence of steps in the docking of DCVs may be similar to that proposed for SVs (Szule et al., 2012). Mobile undocked DCVs rotate, perhaps by Brownian motion, until the appropriate nub-linked transmembrane macromolecules are aligned with booms. They, then, stably connect stepwise to booms, to spars, and together, to ribs and pins, which orients and directs the fusion domain toward the presynaptic membrane (schematized in **Figures 7B,C**).

The connections of SVs with ribs and pins are made ∼15 nm from the presynaptic membrane (Szule et al., 2012). Several lines of evidence (Raciborska et al., 1998; Szule et al., 2012; Jung et al., 2016) make it likely that among other components the ribs and pins connected to SVs contain SNARE proteins while the ribs also contain synaptotagmin. SNARE proteins are known to interact to form a force generating complex that brings the vesicle membrane into stable contact with the presynaptic membrane (Südhof, 2013), the ultimate docking step, while synaptotagmin regulates Ca^2+^-triggered vesicle membrane-presynaptic membrane fusion during synaptic activity (Sudhof, 2013). Our observation that undocked DCVs <15 nm from the presynaptic membrane at active zones are connected to ribs and pins as well as booms and spars, together with the findings of others that DCVs rely on the SNAREs and synaptotagmin for docking and fusion (Voets et al., 2001; Hammarlund et al., 2008; van de Bospoort et al., 2012; Imig et al., 2014; Farina et al., 2015; Kabachinski et al., 2016), supports the concept that not only do the same booms, spars, ribs, and pins that direct the oriented docking and fusion of SVs at neuromuscular junctions also direct the oriented docking and fusion of DCVs but also that this action is mediated by similar molecular mechanisms for both vesicle types.

The dense cores of the DCVs in our samples appeared to consist of an assembly of closely packed, irregularly elongate, interconnected macromolecules. To our knowledge this is the first reference to dense cores being composed of microscopically detectable macromolecules. The macromolecules are likely to include the DCVs’ peptide cargo destined to be released into the synaptic cleft when the DCV membrane fuses with the presynaptic membrane. Such peptides at frog neuromuscular junctions are thought to include neural agrin, calcitonin gene related peptide (CGRP) and neuregulin (Engel, 2008), which are present in axon terminals at neuromuscular junctions across vertebrate species and play different roles in the junctions’ physiology (Matteoli et al., 1988; Hall and Sanes, 1993; Schmidt et al., 2011). We did not undertake in this study to characterize the 3D arrangement of dense core macromolecules as we have done for the luminal assembly of macromolecules in SVs. However, the similarity of the arrangement of nub connection sites on the luminal surface of the DCV membrane to that of SVs raises the possibility that the arrangement of macromolecules in the dense core, to which the nubs are also connected, is stereotypic as is the arrangement of the luminal assembly of macromolecules in SVs. Accordingly, for docked DCVs the orientation of the arrangement would be the same from one docked DCV to the next as it is for the orientation of the luminal assembly in docked SVs.

A stereotypic arrangement of macromolecules and an organized layout of the peptides within it together with a common alignment of the arrangement relative to the presynaptic membrane and the different classes of AZM macromolecules (**Figure 7**) for docked DCVs would favor different peptides, even different portions of the same peptide, entering the synaptic cleft in the same order from one DCV-presynaptic membrane fusion site to the next. The ordered entry of peptides into the synaptic cleft would help explain the functional significance of oriented docking (Harlow et al., 2013). For example, neural agrin is a linear peptide that is ∼90 nm long when fully extended (Denzer et al., 1998). Upon entry into the synaptic cleft it binds at several different sites along its length to different components of the highly organized network of glycoproteins that constitute the synaptic cleft basal lamina. At a site near its C-terminus agrin binds to the peptides Lrp4 and MuSK, which project from the postsynaptic plasma membrane of the muscle fiber into the basal lamina; this interaction directs the formation and maintenance of the muscle fiber’s postsynaptic apparatus, which is essential for neurotransmitter-mediated synaptic impulse transmission (McMahan, 1990; Tintignac et al., 2015). An orderly entry of agrin’s different binding sites into the synaptic cleft might be essential for the sites to reach their respective basal lamina targets. AZM-mediated oriented docking of DCVs is likely to occur at the neuromuscular junctions of species other than frog. Genetic insertion of histochemically detectable tags at specific sites along peptides (e.g., Shu et al., 2011), together with mapping the arrangement of the sites in the core of DCVs and in the synaptic cleft by electron tomography may be useful for understanding the layout of the peptides within the core and the steps and mechanisms involved in their exocytosis and translocation to their targets.

## Author Contributions

JJ, JS, and UM designed the experiments, collected and analyzed the data, and wrote the manuscript. KS analyzed the data. RM prepared tissue and collected the data.

## Conflict of Interest Statement

The authors declare that the research was conducted in the absence of any commercial or financial relationships that could be construed as a potential conflict of interest. The handling Editor and reviewer RW declared their involvement as co-editors in the Research Topic, and confirm the absence of any other collaboration.

## Footnotes

1 em3d.org
